# Impact of a Digital Decision Aid When Choosing Between Face-to-Face and Guided Internet-Based Psychological Interventions for Depression Among Chinese-Speaking Participants in Hong Kong: Randomized Controlled Trial

**DOI:** 10.2196/54727

**Published:** 2025-05-06

**Authors:** Larry Auyeung, Winnie WS Mak, Ella Zoe Tsang, Philo Liu Yang

**Affiliations:** 1 School of Arts and Humanities Tung Wah College Kowloon China (Hong Kong); 2 Department of Psychology Chinese University of Hong Kong Shatin, New Territories China (Hong Kong)

**Keywords:** decision aid, shared decision-making, guided internet-based psychological interventions, ICBT, depression

## Abstract

**Background:**

The expansion of e-mental health services offers diverse treatment options. As the variety of available interventions grows, helping individuals navigate these options effectively becomes essential.

**Objective:**

This study evaluates the effects of a decision aid for users when choosing between guided internet-based psychological interventions and in-person psychotherapy.

**Methods:**

A web-based, randomized controlled trial was conducted with 148 Chinese-speaking adult participants from Hong Kong with Patient Health Questionnaire-9 (PHQ-9) scores ≥10 (indicating clinical depression). Participants were recruited by electronic direct mail, social media, university mass mail, and online advertising then randomly assigned to either the decision aid intervention group or the attention control group. The study’s assessments were conducted online through self-administered questionnaires before and after the intervention, while the intervention was delivered via Zoom. The decision aid group underwent a brief interactive, self-directed, web-based decision aid. The decision aid included psychoeducation on depression and treatments, a comparison between internet-based interventions and face-to-face therapy, and personalized reports for value clarification. The attention control involved an unguided web search on mental health information. Primary outcome measures included decision conflict (measured using the SURE tool and Decision Conflict Scale), while secondary outcomes included stage of decision-making, satisfaction with decision, perceived benefits and risks, and likelihood of service utilization.

**Results:**

Time-by-intervention interactions in ANOVA were found, which indicated that the reduction in decisional conflict was more significant in the decision aid group than in the control group, as measured using the brief SURE tool (*F*_1,145_=6.47, *P=*.01; partial η^2^ = 0.043; 95% CI 0.002-0.122) and decision conflict scale (*F*_1,136_=9.56, *P*=.002; 95% CI 0.0086-0.16). Specifically, interaction effects were observed for 3 of the 5 decision conflict subscales: The decision aid group reported feeling more “informed,” experiencing greater “support,” and being better able to make “effective decisions.” Participants in the decision aid group also reported more advanced stages of decision-making; however, a significant difference between groups was not found for satisfaction with the decision. Although there was no significant change in perceived benefit, participants in the decision aid group had significantly greater reductions in their perceived risks associated with a guided internet-based psychological intervention. In addition, participants who used the decision aid were 2.26 times more likely to prefer (odds ratio [OR] 2.26, 95% CI 1.11-4.60; *P=*.02) and 2.53 times more likely to use (OR 2.53, 95% CI 1.13-4.92; *P=*.006) a guided internet-based psychological intervention than participants who searched for mental health information on the web by themselves.

**Conclusions:**

This study demonstrates the extent of the utility and value of a decision aid for assisting individuals with depressive symptoms make informed choices related to e-mental health. Decision aids may facilitate the uptake of digital mental health services. Future research should explore the behavioral and long-term impact and generalizability of decision aids in applied settings.

**Trial Registration:**

Chinese Clinical Trial Register ChiCTR2300077323; https://tinyurl.com/2n34ea69; ClinicalTrials.gov NCT05477420; https://clinicaltrials.gov/study/NCT05477420

## Introduction

### e-Mental Health and Its Potential for Treating Depression

Depression is prevalent and a leading cause of disability, mortality, loss of productivity, and health care expenditure [1]. Psychological interventions not only are effective for the treatment of depression [2] but are also considered a ﬁrst-line treatment for major depression [3]. Psychological interventions are also the preferred treatment method for most people with depression [4]. Despite the effectiveness [5] and preferences for psychological interventions, overall service utilization by people with depression remains low, with rates from less than 4.8% to 12.8% in various age subgroups in the population [6]. The reasons for suboptimal use might be attributed to the exorbitant expense; extended commute time; need for weekly hour-long therapy sessions, which may clash with the therapist’s timetable; and obstacles on the supply side, including lengthy waiting periods in the public health care sector. Obtaining sick leave for psychological appointments can also be challenging due to the prevailing work culture and stigmatized perception toward taking medical leave for mental health services [7]. A potential solution to overcoming these barriers is providing treatment digitally, such as via an online platform or mobile apps [8]. Notably, e-mental health services have demonstrated comparable effectiveness to face-to-face counterparts [9].

e-Mental health services have also been implemented globally. For example, in the United Kingdom, there are 2 computerized interventions for mental health conditions recommended in the clinical treatment guidelines (ie, Beating the Blues for depression [10] and Silver Cloud [11]). During COVID-19, the implementation of eHealth services also accelerated in Australia, with uptake of online cognitive behavioral therapy for anxiety and depression increasing 5-fold during the pandemic [12]. In Hong Kong, although the implementation process of e-mental health services is considered slower than that in Western countries; although no internet-based intervention for depression has been implemented or formally endorsed in Hong Kong’s public health care system, an e-mental health service has been informally integrated in the Hong Kong health care system. For example, two philanthropic trust–funded, technology-mediated, guided, self-help platforms have been established and implemented by local universities over the past 3 years, including the “Jockey Club Tour Heart+ Project” [13] and “CANDO” [14].

With the continuous effort to establish and disseminate e-mental health services, it is important to address potential concerns, such as the presence of decision conflict when choosing between a guided internet-based psychological intervention and face-to-face psychotherapy, prior to the full implementation stage of e-mental health services [15].

### The Importance of User Preference and Acceptability in e-Mental Health Services

For successful implementation of e-mental health services, users’ preferences and acceptability cannot be neglected. Treatment acceptability has been framed as a key factor for successful dissemination and implementation of any new health service because a treatment could be clinically effective yet unacceptable for users [16,17].

Based on both frameworks for technology adoption and previous research [18-20], acceptability was defined in this study as the perception of the service (eg, perceived benefits and risks or side effects of using the service), and intentions to use the services were used as indicators for public acceptability [16,17]. Furthermore, users’ preferences and acceptability not only influence satisfaction but also have significant implications for adherence and outcomes [21]. According to a meta-analysis of various treatment formats, individuals who were matched with their preferred treatment had a higher chance of showing improvement and were almost one-half as likely to drop out of treatment compared with those whose preferred choice of treatment was not offered [22]. Previous studies also suggest that preferences and acceptability are significantly related to important processes and outcomes of treatment including service initiation, adherence, compliance, engagement, and the development of a working alliance [21].

### The Applicability of Decision Aids for Clarifying Preferences for Psychological Interventions

In the realm of researching users’ preferences for psychological treatments for depression, an important and understudied aspect is the role of decision aids in decision conflict and treatment uptake. Decision aids are defined as tools that assist patients and service users with understanding the decision at hand and provide information on service options and their respective outcomes. Additionally, decision aids encourage service users to express their own values and preferences related to these options and make informed decisions [23].

In the last decade, active participation of service users and patients in the decision-making process regarding their health care has been increasingly advocated [24]. In line with this advocacy, decision aids are specifically designed to promote and facilitate shared decision-making [25], which is widely recognized as the crux of patient-centered care, by providing users with the necessary information and support to make informed choices. Decision aids could be developed in different formats (eg, paper and pen instruments, videos, audio, websites, interactive software) [26]. Decision aids can be used alone by the users or in collaboration with service providers such as nurses and physicians. Typically, they include explanations about treatment options, descriptions of the benefits and harms supported by scientific evidence, and information about the characteristics of health services based on local situations [27]. Recent systematic reviews show that decision aids are effective at improving individuals’ knowledge about available treatments and reduce decisional conflict, such as uncertainty about the course of action to take. Decision aids have also been shown to reduce the proportion of people who were passive and undecisive in decision-making [23].

### Research Gaps

In the area of treatment for depression, results show that a majority of people with depression consider it important to receive information about their available treatment options and to participate in shared decision-making [28,29]. However, findings show that people with depression often perceive less involvement in decision-making than they desire [30,31]. Moreover, decision aids are being widely and successfully adopted for many physical health conditions (such as breast cancer treatment [32], HIV preexposure prophylaxis [33], colon cancer screening [34], and smoking cessation [35]) but not for depression, despite this unmet demand.

Practically, the study of decision aids in digital mental health services is particularly timely, as the pandemic has provided an opportunity to expedite the development of eHealth services [12]. It is now crucial for the academic community to proactively address challenges in service dissemination [36]. In the post-COVID-19 era, individuals are increasingly familiar with remote services and may consider digital psychological interventions as a viable option when seeking assistance [37]. However, when service users feel uncertain about the most suitable service for their needs, a decision aid may serve as a valuable tool to bridge the treatment gap through shared decision-making.

### Study Goals, Hypotheses, and Objectives

This study aimed to investigate whether a brief digital decision aid is effective at reducing decisional conflict, thereby supporting the decision-making process of people with depression. Notably, a guided internet-based psychological intervention was chosen as the focus of this study because it is recommended as a front-line treatment for mild to moderate depression by the National Institute for Health and Care Excellence (NICE) guidelines and is the preferred treatment modality before face-to-face therapy and medication due to its lower level of invasiveness and greater cost-effectiveness [38].

The study’s primary hypothesis was that the decision aid will reduce decision conflict, which is defined as individual perceptions of uncertainty about which course of action to take in a health circumstance [39].

The study’s secondary hypothesis was that the decision aid would facilitate decision-making stages [40] and improve decision satisfaction [41]. Furthermore, we hypothesized that the decision aid would enhance individuals’ perceptions of guided internet-based psychological interventions, which may be perceived as a less useful and potentially riskier treatment option due to unfamiliarity [42].

## Methods

### Study Design and Procedures

This study was conducted as an online, randomized controlled trial assessing the effects of a decision aid compared with a control condition. The assessment was conducted online through self-administered questionnaires via Qualtrics, while the intervention was delivered via Zoom by research assistants. Participants were recruited by electronic direct mail, social media, university mass mail, and online advertising. Participants who were 18 years of age or older, were Chinese-speaking, and had a Patient Health Questionnaire-9 (PHQ-9) score [43] ≥10, indicating diagnostic depression [44], were included in the study.

Eligible participants who had computer access provided informed consent by acknowledging that they had read the information statement by clicking a box then clicking a separate box to indicate that they consented to participate in the research. Following self-administered registration and online screening using the PHQ-9 score, participants were randomized to the decision aid or control group. Participants were then invited by the research assistant to complete an online, self-administered, pre-intervention questionnaire and an online, self-administered, postintervention questionnaire that focused on outcome measures immediately after the intervention. Time spent on the decision aid was recorded.

### Ethical Considerations

All participants provided informed consent voluntarily and had the right to withdraw at any time, and the data were de-identified and encrypted. Only members of the research team were permitted to access the data. The study was approved by the ethics committees of the Chinese University of Hong Kong (SBRE-21-0325) and retrospectively registered. In fact, we prospectively registered the decision aid study along with another related survey study. However, during the publication phase, we discovered an oversight in our research assistant’s entry of the registration information, which did not include the outcome variables for the decision aid but mistakenly entered the outcomes that actually pertained to the survey study. Since then, we corrected the prospective registration, and the additional retrospective registration was created for the decision aid study on November 6, 2023. We understand that the primary purpose of registration is to prevent biased reporting. Therefore, we have reported all findings—both significant (eg, decision conflict) and nonsignificant (eg, decision satisfaction)—regardless of whether they aligned with or contradicted our hypothesis. Additionally, we made an effort to discuss all findings thoroughly in the main text, acknowledging the limitations of the decision aid and ensuring that both favorable and unfavorable outcomes are disclosed to the readers. HK $50 was provided to participants as a token of thanks upon completion of the study.

### Intervention Group: A Self-Help, Interactive, Web-Based Decision Aid for a Psychological Intervention

**Figure 1:**
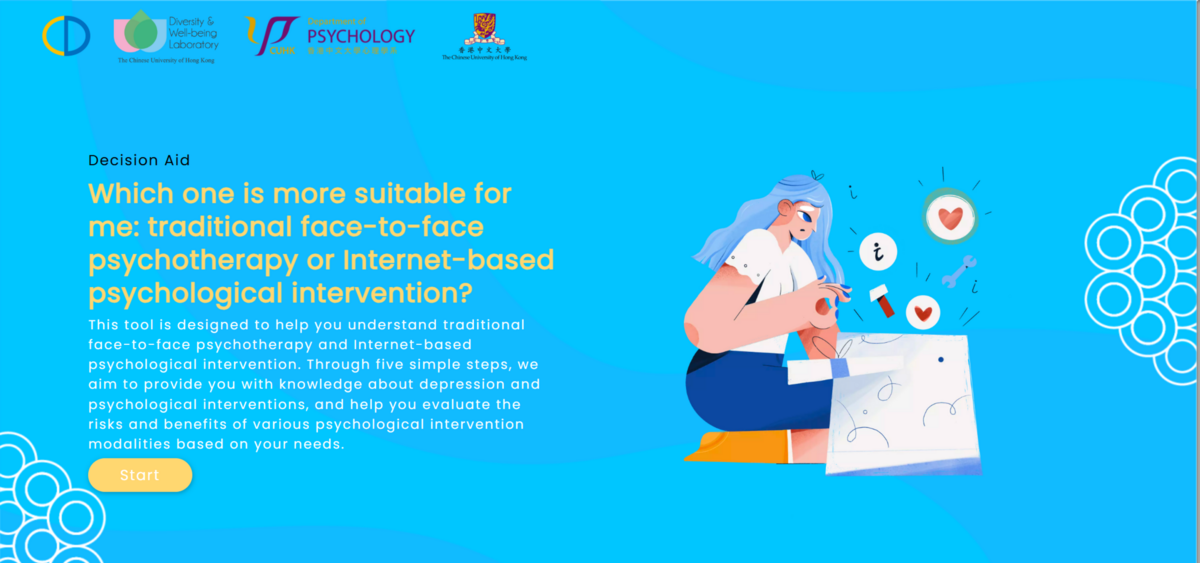
Self-help, interactive, web-based decision aid (DA) for a psychological intervention.

**Figure 2:**
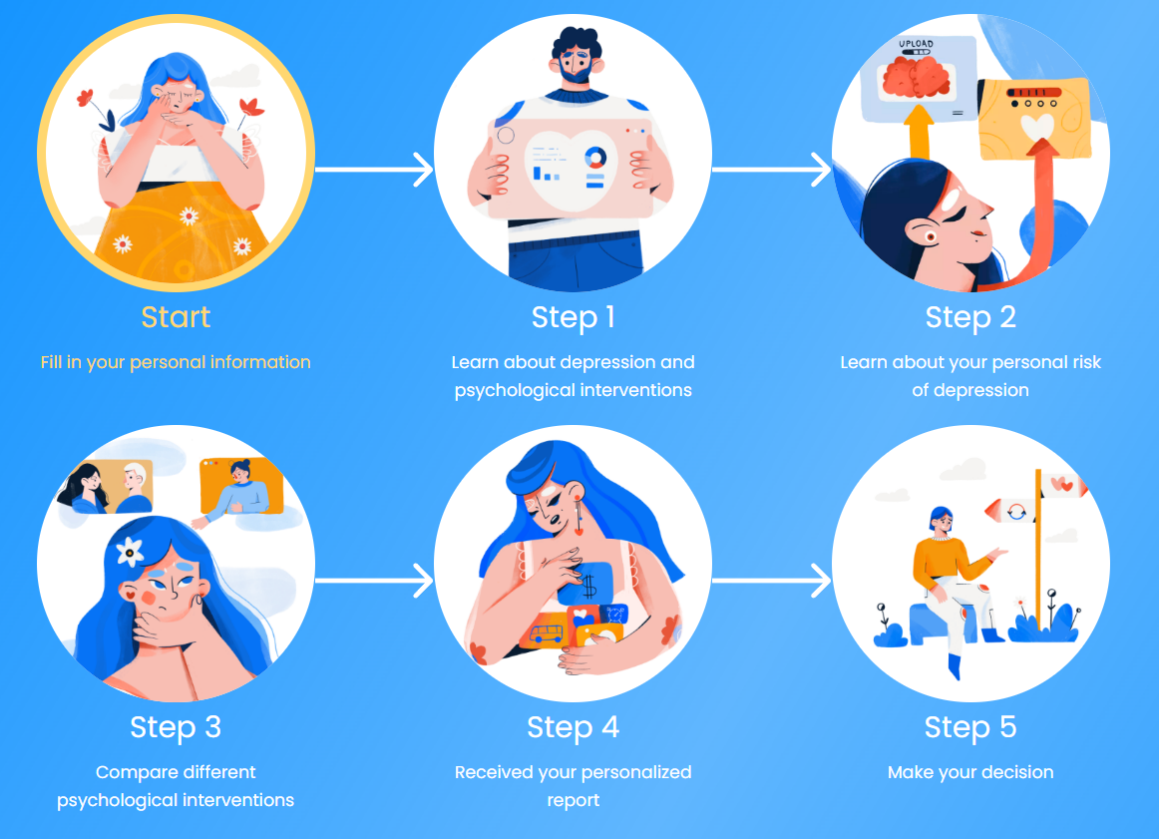
Journey map of the decision aid.

The decision aid group was asked to spend 15 minutes using a self-help, interactive, web-based decision aid on psychological interventions. The decision aid was developed by the study’s author and a professor of clinical psychology. They used existing literature and local data, including information on the cost and waiting time for psychological interventions, as references during the development process. The materials were made available online by a web developer in the author’s affiliated laboratory (Figures 1 and 2).

The decision aid consisted of several components, including psychoeducation on depression and its treatments, an assessment of depressive symptoms, a comparison between internet-based psychological interventions and traditional face-to-face psychotherapy, personalized reports on value clarification and preference elicitation, and information on common inquiries regarding psychological interventions, which included:

- How do the treatment options work?
- Who will be providing the services?
- How much do different options cost?
- Where do different options take place?
- How long and how frequently will the psychological intervention be?
- Will the presented options work?
- How long is the waiting period?
- Will there be any risks or unintended harm? Is there anything that users need to be concerned about?

Each of these questions was displayed on a new web page, individuals were asked to rate the importance of each service attribute corresponding to the question, and their preferences were elicited. At the end of the decision aid, individuals were presented with an individualized report based on the assigned importance of each question, which could guide them in selecting the most suitable form of psychological intervention for their needs. Decision aid content in the participants’ local languages could be found at the authors’ affiliated laboratory website and is available upon request [45].

### Control Group: Unguided Usual Search Strategies for Online Mental Health Information

As the internet has increasingly become the key source of information about health and is being used as a supplement to traditional sources of health information [46], the enormous volume of online health information has revolutionized service users’ education [47]. Moreover, people with a stigmatized health problem, such as depression, are more inclined to use the internet as a tool to seek related information than people with less-stigmatized health problems [48,49]. In this study, the control group was instructed to spend 15 minutes using their typical strategies to search for mental health information using keywords such as “depression” or/and “depression treatment” via any search engine in an unguided format. The time limit was set so that the instruction for the duration of the experimental activity was matched across groups.

### Measures

#### Baseline and Screening Measures

Sociodemographic variables including gender, age, perceived socioeconomic status, and marital status were collected. Depression severity was measured using the PHQ-9, which provides a brief measure of current depression symptoms (past 2 weeks) using a 4-point Likert scale ranging from 0 (not at all) to 3 (nearly every day).

#### Outcome Measures

##### Decisional Conflict

Decisional conflict was measured using the brief SURE tool [50]and Decisional Conflict Scale (DCS) [39]. SURE was used to assess individuals’ perceptions of feeling sure, informed, supported, and clear about what matters most. Sample items include “Do you feel SURE about the best choice for you?” The DCS measures 5 dimensions of decision-making (feeling uncertain, uninformed, unclear about values, unsupported, ineffective decision-making). The scale consists of 16 questions using a 5-point Likert scale ranging from 1 (strongly agree) to 5 (strongly disagree). Sample items include “I have enough advice to make a choice.”

##### Stage of Decision-Making Scale

The Stage of Decision-Making Scale [51] measures individuals’ readiness to engage in decision-making. It consists of a single item with 6 response options from “haven't started to think about the choices” to “have already made a decision and am unlikely to change my mind.” Earlier stages of decision-making are associated with higher levels of decisional conflict and vice versa.

##### Satisfaction With Decision Scale

The Satisfaction With Decision scale is a 6-item Likert scale rated from 1 (strongly disagree) to 5 (strongly agree) to assess satisfaction with the decision, with higher scores indicating higher levels of satisfaction. Sample items include “I am satisfied that I am adequately informed about the issues important to my decision” [41].

##### Perceived Benefits and Risks

Participants were asked to rate the degree to which they perceived face-to-face psychotherapy and guided internet-based psychological intervention to be effective by indicating their estimation of the proportion (0%-100%) of people with depression who experience a clinically significant improvement in depressive symptoms after receiving the corresponding services and their estimation of the proportion (0%-100%) of people with depression who experience a negative effect on depressive symptoms after receiving the corresponding services.

##### Service Preference Identity

Two items were used to assess participants’ service preference identities, with one item assessing personal liking and the other item assessing likelihood of use. Participants were asked to indicate their preference (liking/favor) for either traditional face-to-face or guided internet-based psychological interventions as their “preferred treatment option.“ An ”unsure“ option was included. Participants who chose the guided internet-based psychological intervention in this item were classified as an “e-preferrer.” Participants were then asked to indicate their preference between traditional face-to-face psychotherapy or a guided internet-based psychological intervention as a treatment option they would be “likely to use.” Again, an “unsure” option was included. Participants who selected the guided internet-based psychological intervention in this item were classified as “e-service–inclined individuals.”

## Results

### Sociodemographic Information

**Figure 3:**
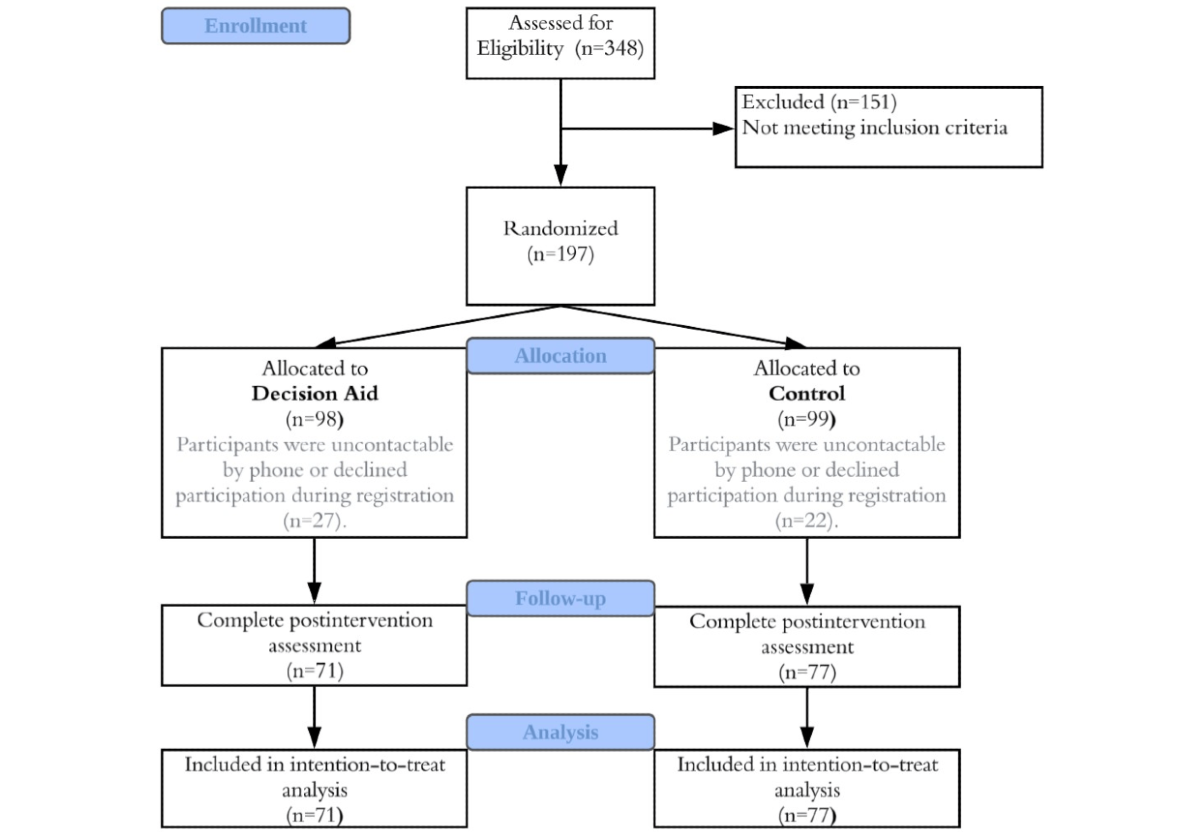
Study flowchart.

Baseline characteristics are presented in Table 1. A total of 148 participants completed the study (Figure 3). The participants’ mean age was 28.35 (SD 7.74) years, and their ages ranged from 20 years to 65 years. Participants were predominantly female (111/148, 75%), and most of them had attained at least a bachelor’s degree (132/148, 89.2%). Participants placed themselves round the middle (mean 4.91, SD 1.67) of the socioeconomic ladder, with 10 ladder rungs ranging from 1 (bottom) to 10 (top). Of the participants, 41.2% (61/148) had received mental health treatment before. Regarding PHQ-9 scores, 41.9% (62/148) of the participants were classified as having moderate depression (PHQ-9: 10-14), and 57.4% (85/148) were classified as having severe depression (PHQ-9: ≥15). Participants in the decision aid group were not significantly different from the control group in age (t_145_=0.48, *P*=.63), gender (χ^2^_2_=2.02, *P*=.36), marital status (χ^2^_4_=4.32, *P*=.37), employment (χ^2^_4_=3.37, *P*=.99), education (χ^2^_4_=5.05, *P*=.28), perceived socioeconomic status (t_144_=–0.969, *P*=.33), history of mental health treatment (χ^2^_1_=3.98, *P*=.07), and severity of depression (χ^2^_1_=0.712, *P*=.41).

**Table 1:** Sociodemographic data at baseline.

Characteristic			Decision aid group (n=71)		Control group (n=77)		All participants (N=148)		Test statistics (*df*)^a^			*P* value	
Age (years), mean (SD)			28.67 (7.28)		28.05 (8.17)		28.35 (7.74)		0.48 (145)^b^			.63	
**Gender, n (%)**										2.02 (2)^c^			.36
	Men	19 (26.8)		16 (20.8)		35 (23.6)							
	Women	50 (70.4)		61 (79.2)		111 (75)							
	Nonbinary	1 (1.4)		0 (0)		1 (0.7)							
	Not disclosed	0 (0)		0 (0)		0 (0)							
**Marital status, n (%)**										4.32 (4)^c^			.37
	Single	50 (70.4)		58 (75.3)		108 (73)							
	Unmarried	11 (15.5)		10 (13)		21 (14.2)							
	Cohabitating	0 (0)		2 (2.6)		2 (1.4)							
	Married	7 (9.9)		7 (9.1)		14 (9.5)							
	Divorced	2 (2.8)		0 (0)		2 (1.4)							
	Widowed	0 (0)		0 (0)		0 (0)							
**Employment, n (%)**										3.37 (4)^c^			.99
	Part-time/student	39 (54.9)		45 (58.4)		84 (56.8)							
	Full-time worker	25 (35.2)		27 (35.1)		52 (35.1)							
	Unemployed/retired	3 (4.2)		2 (2.6)		5 (3.4)							
	Houseperson/caregiver	1 (1.4)		1 (1.3)		2 (1.4)							
	Other	1 (1.4)		1 (1.3)		2 (1.4)							
**Education, n (%)**										5.05 (4)^c^			.28
	Higher secondary school	2 (2.8)		2 (2.6)		4 (2.7)							
	Diploma/community college/associate degree	8 (11.3)		2 (2.6)		10 (6.8)							
	Bachelor’s degree	50 (70.4)		59 (76.6)		109 (73.6)							
	Master’s degree	8 (11.3)		12 (15.6)		20 (13.5)							
	Doctorate	1 (1.4)		2 (2.6)		3 (2)							
Perceived socioeconomic status (1-10), mean (SD)			4.77 (1.80)		5.04 (1.58)		4.91 (1.67)		–0.969 (144)^b^			.33	
**History of psychological treatment, n (%)**										3.98 (1)^c^			.07
	Yes	35 (49.3)		26 (33.8)		61 (41.2)							
	No	35 (49.3)		51 (66.2)		86 (58.1)							
**Depression (PHQ-9^d^), n (%)**										0.712 (1)^c^			.41
	10-14 (moderate)	27 (38)		35 (45.5)		62 (41.9)							
	15 (severe)	43 (60.6)		42 (54.5)		85 (57.4)							

^a^Comparison between the decision aid and control groups.

^b^*t* test.

^c^Chi-square test.

^d^PHQ-9: Patient Health Questionnaire-9.

### Primary Outcomes: Decision Conflict

#### SURE Test

A repeated measures ANOVA was conducted, and the time-by-intervention results can be found in Table 2. The findings indicated that, using the brief SURE test items, the web-based decision aid resulted in significantly greater improvements in effective and informed decision-making (*F*_1,145_=6.47, *P=*.01; partial η^2^=0.043; 95% CI 0.002-0.122) than the control. Specifically, the chi-square tests indicated that, compared with the control group, a significantly greater percentage of the participants in the decision aid group agreed that they “know the benefits and harms of each option” (decision aid: 67/71, 94%; control: 59/76, 78%; χ^2^_1_=8.40) and “had enough support and advice to make a choice” (decision aid: 57/71, 80%; control: 45/77, 58%; χ^2^_1_ =8.23) after Bonferroni correction. However, the differences in the percentage of participants who agreed that they “feel SURE about best choice” and were “clear about which benefits and risks matter most” were not significant.

**Table 2:** The effect of a web-based decision aid on the decision-making process.

Measures by period and group					Results		Test statistics (*df*)			Partial η^2^ (Cohen *d*)			*P* value						Bonferroni-corrected *P* value	
**SURE test, mean (SD)**								6.47 (1, 145)^a^			0.043 (0.87)			.01						—^b^
	**Pre-intervention**																			
			Control group	5.71 (1.60)																
			Decision aid group	5.61 (1.63)																
	**Postintervention**																			
			Control group	5.24 (1.46)																
			Decision aid group	4.56 (0.94)																
**Feels SURE about best choice, n (%)**																				
	**Pre-intervention**						0.87 (1)^c^			—			.40			—				
			Control group (n=77)	43 (56)																
			Decision aid group (n=71)	45 (63)																
	**Postintervention**						3.53 (1)^c^			—			.07			.28				
			Control group (n=76)	50 (66)																
			Decision aid group (n=71)	56 (79)																
**Knows the benefits and harms of each option, n (%)**																				
	**Pre-intervention**						0.33 (1)^c^			—			.62			—				
			Control group (n=77)	48 (62)																
			Decision aid group (n=71)	41 (58)																
	**Postintervention**						8.40 (1)^c^			—			.004			.02				
			Control group (n=76)	59 (78)																
			Decision aid group (n=71)	67 (94)																
**Clear about which benefits and risks matter most, n (%)**																				
	**Pre-intervention**						0.017 (1)^c^			—			>.99			—				
			Control group (n=77)	48 (62)																
			Decision aid group (n=71)	45 (63)																
	**Postintervention**						4.81 (1)^c^			—			.03			.12				
			Control group (n=77)	59 (77)																
			Decision aid group (n=71)	64 (90)																
**Has enough support and advice to make a choice, n (%)**																				
	**Pre-intervention**						0.27 (1)^c^			—			.63			—				
			Control group (n=77)	39 (51)																
			Decision aid group (n=71)	39 (55)																
	**Postintervention**						8.23 (1)^c^			—			.005			.02				
			Control group (n=77)	45 (58)																
			Decision aid group (n=71)	57 (80)																
**DCS^d^, mean (SD)**								9.56 (1, 136)^a^			0.066 (1.39)			.002			—			
	**Pre-intervention**																			
			Control group	42.87 (9.71)																
			Decision aid group	40.26 (10.10)																
	**Postintervention**																			
			Control group	37.69 (11.82)																
			Decision aid group	30.50 (9.33)																
**DCS: Uncertainty, mean (SD)**								3.35 (1, 140)^a^			0.023 (0.54)			.07			.35			
	**Pre-intervention**																			
			Control group	8.59 (2.50)																
			Decision aid group	8.21 (2.73)																
	**Postintervention**																			
			Control group	7.62 (2.70)																
			Decision aid group	6.59 (2.73)																
**DCS: Informed, mean (SD)**								8.48 (1, 143)^a^			0.056 (0.24)			.004			.02			
	**Pre-intervention**																			
			Control group	7.84 (2.39)								.								
			Decision aid group	7.50 (2.28)																
	**Postintervention**																			
			Control group	6.47 (2.27)																
			Decision aid group	4.97 (1.61)																
**DCS: Values clarity, mean (SD)**								4.36 (1, 145)^a^			0.030 (0.18)			.04			.20			
	**Pre-intervention**																			
			Control group	7.92 (2.66)																
			Decision aid group	7.21 (2.58)																
	**Postintervention**																			
			Control group	6.81 (2.66)																
			Decision aid group	5.30 (1.68)																
**DCS: Support, mean (SD)**								6.89 (1, 146)^a^			0.045 (0.22)			.01			.05			
	**Pre-intervention**																			
			Control group	8.34 (2.34)																
			Decision aid group	8.17 (2.55)																
	**Postintervention**																			
			Control group	7.40 (2.43)																
			Decision aid group	6.32 (2.48)																
**DCS: Effective decision, mean (SD)**								8.51 (1, 145)^a^			0.055 (0.24)			.004			.02			
	**Pre-intervention**																			
			Control group	10.08 (2.53)																
			Decision aid group	9.61 (2.57)																
	**Postintervention**																			
			Control group	9.39 (3.23)																
			Decision aid group	7.75 (2.88)																
**Stage of Decision-Making, mean (SD)**								5.14 (1, 146)^a^			0.034 (0.19)			.03			—			
	**Pre-intervention**																			
			Control group	2.83 (1.56)																
			Decision aid group	2.90 (1.51)																
	**Postintervention**																			
			Control group	3.05 (1.44)																
			Decision aid group	3.55 (1.48)																
**Satisfaction With Decision scale, mean (SD)**								0.21 (1, 145)^a^			0.001 (0.03)			.65			—			
	**Pre-intervention**																			
			Control group	21.22 (3.03)																
			Decision aid group	21.37 (3.61)																
	**Postintervention**																			
			Control group	22.54 (3.98)																
			Decision aid group	22.97 (5.08)																

^a^ANOVA.

^b^Not applicable.

^c^Chi-square test.

^d^DCS: Decision Conflict Scale.

#### Decision Conflict Scale (DCS)

The findings showed that decisional conflict was reduced significantly more in the decision aid group than in the control group, as indicated by a time-by-intervention interaction (*F*_1,136_=9.56, *P*=.002; partial η^2^=0.066; 95% CI 0.0086-0.16). It should be noted that, although there was a significant main effect for time (*F*_1,136_=101.8, *P=*.002; partial η^2^=0.43; 95% CI 0.30-0.53), indicating that both the decision aid and control groups had less decisional conflict in their decision-making from pre- to postintervention, the change in decision conflict was significantly greater in the decision aid group. In addition, significant interaction effects were found for 3 of the 5 DCS subscales: “informed,” “support,” and “effective decision.”

### Secondary Outcomes

#### Stage of Decision-Making and Satisfaction With Decision Scales

Regarding the stage of decision-making, participants in the decision aid group reported more advanced stages of decision-making (*F*_1,146_=5.14, *P=*.03; partial η^2^=0.034; 95% CI 1.44^-05^ to 0.11). Results of the binary logistic regression analysis indicated that participants in the decision aid group were more than 2 times (odds ratio [OR] 2.27, 95% CI 1.16-4.43; *P=*.02) as likely as those who were allocated to the control group to be in the latter 3 stages of decision-making. As for satisfaction with decision, no significant difference between the 2 groups was found.

#### Perceived Benefits and Risks and Preference Identity

Using repeated measures ANOVA, the time-by-intervention indicated that there was no significant group difference in the changes of perceived benefits associated with a guided internet-based psychological intervention (*F*_1,145_=3.48, *P=*.06; partial η^2^=0.023) and no significant group difference in the changes of perceived benefits associated with face-to-face psychotherapy (*F*_1,145_=1.89, *P=*.17; partial η^2^=0.013; Table 3). Thus, participants in the decision aid group were not significantly more likely than participants in the control group to observe an increase in the perceived benefits of both a guided internet-based psychological intervention and face-to-face psychotherapy.

However, attitudes on the perceived benefit of psychotherapy did not shift differentially across groups, a group difference in the change in attitude in terms of perceived risks associated with a guided internet-based psychological intervention was observed. Compared with participants in the control group, participants in the decision aid group experienced a significantly greater reduction in their perceived risks associated with a guided internet-based psychological intervention (*F*_1,145_=8.04, *P=*.005; partial η^2^=0.055; 95% CI 0.004-0.14). However, there was no evidence for a comparative advantage of using a decision aid for reducing the perceived risk associated with face-to-face psychotherapy (*F*_1,145_=2.40, *P=*.12; partial η^2^=0.018).

Regarding service preference identity, as indicated by the logistic regression analysis, the odds of being an e- (participants who preferred a guided internet-based psychological intervention) was 2.26 times greater preferrer for participants who used the decision aid than participants who searched for mental health information on the web by themselves (OR 2.26, 95% CI 1.11-4.60; *P*=.02). Moreover, the odds of being an e-service–inclined user (participants who were more likely to use a guided internet-based psychological intervention) was 2.53 times greater for participants who used a decision aid than participants who searched for mental health information on the web by themselves (OR 2.53, 95% CI 1.13-4.92; *P*=.006).

**Table 3:** Shift in attitudes and preferences after using a decision aid.

Measures by period and group					Results, mean (SD)		*F* statistic (*df*)^a^	Partial η^2^ (Cohen *d*)		*P* value
**Perceived benefit associated with:**										
	**Guided internet-based psychological intervention**						3.48 (1, 145)	0.023 (0.15)		.06
		**Pre-intervention**								
			Control group	53.54 (16.20)						
			Decision aid group	49.93 (17.52)						
		**Postintervention**								
			Control group	59.17 (17.30)						
			Decision aid group	60.59 (20.43)						
	**Face-to-face psychotherapy**						1.89 (1, 145)	0.013 (0.11)		.17
		**Pre-intervention**								
			Control group	70.95 (14.80)						
			Decision aid group	65.29 (16.19)						
		**Postintervention**								
			Control group	74.40 (16.44)						
			Decision aid group	65.54 (18.48)						
**Perceived risk associated with:**										
	**Guided internet-based psychological intervention**						8.04 (1, 145)	0.055 (0.24)		.005
		**Pre-intervention**								
			Control group	36.14 (21.25)						
			Decision aid group	34.33 (16.81)						
		**Postintervention**								
			Control group	32.55 (22.72)						
			Decision aid group	22.55 (19.87)						
	**Face-to-face psychotherapy**						2.40 (1, 145)	0.018 (0.13)		.12
		**Pre-intervention**								
			Control group	34.77 (21.84)						
			Decision aid group	34.77 (17.77)						
		**Postintervention**								
			Control group	31.25 (23.65)						
			Decision aid group	26.68 (21.72)						

^a^group × time.

### The Effect of Duration of Use and Severity of Depression on Reduction of Decision Conflict

The mean time taken for the decision aid was 6.85 minutes, with a range between 1.85 minutes and 15.8 minutes, and 58 of 71 (82%) participants finished using the decision aid within 10 minutes.

**Figure 4:**
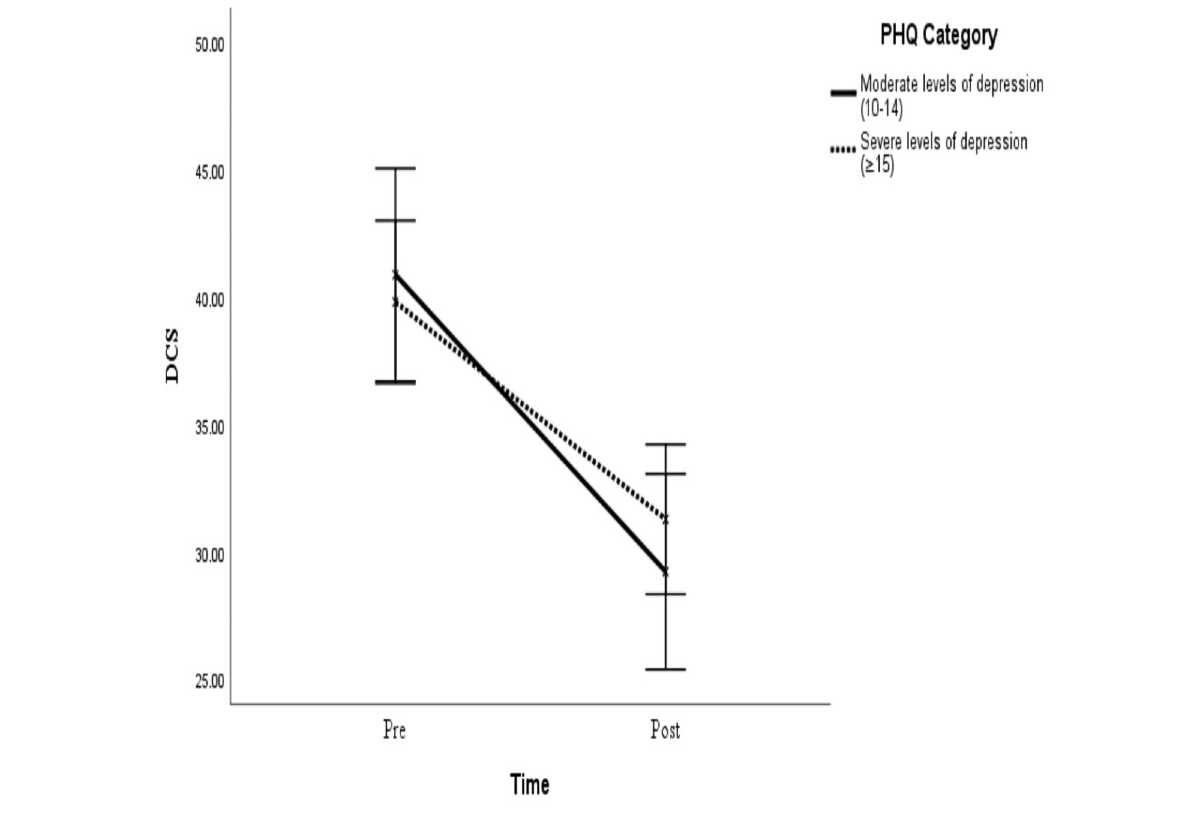
Comparison of pre-intervention (Pre) and postintervention (Post) decision conflict among people with moderate and severe levels of depression. DCS: Decisional Conflict Scale; PHQ: Patient Health Questionnaire. Error bars: 95% CIs.

Notably, the duration of use did not have a significant effect on the reduction of decision conflict (*F*_1,59_=1.49, *P=*.35). The severity of depression also did not have a significant effect on the reduction of decision conflict (*F*_1,133_=.282, *P=*.60) nor a 3-way interaction effect with the time by group allocation (*F*_1,133_=2.35, *P=*.13), indicating the applicability of a decision aid among people with moderate or severe levels of depressive symptoms (Figure 4).

## Discussion

### Principal Findings

This study aimed to investigate the ability of a web-based decision aid to improve decision-making and reduce decisional conflict among individuals with depression. The results of our study revealed several important findings.

First, use of the decision aid was associated with a significant improvement in decision conflict and advancing individuals to more advanced stages of decision-making. Although decision aids are predominantly used in a medical context [52,53], this finding is in line with previous research that has highlighted the potential utility of decision aids for enhancing users' decision-making when choosing psychological treatment [28,54]. The results of this study align with those of a previous usability study that found both clinicians and patients with cancer strongly endorsed the use of a decision aid for treatment decisions related to anxiety or depression [55]. However, although previous studies highlighted the potential utility of decision aids in the mental health care context, the study by Rayner et al [55] focused on psychotherapy in general even when preference for self-help was one of the reasons for declining help stated by cancer patients in distress [56], and many other studies focused on psychotropic medications [52]. In contrast, this study included a guided internet-based psychological intervention as an alternative option, which is in line with the NICE guidelines, which explicitly state that service providers should consider the least intrusive and least resource-intensive treatment first [38]. More importantly, this study adopted a robust experimental design to distinguish the effect of a decision aid from self-searching for mental health information. Studies have found the internet to be one of the preferred methods for accessing mental health information [57], with 61.6% of youths reporting that they had used the internet to search for mental health information while 82.9% expressed that they are likely to use informational websites if experiencing challenging times [58]. This distinction is crucial for demonstrating the added value of a decision aid based on an ecologically valid comparison. However, although the decision aid in this study guided participants to a later stage of decision-making, it did not lead to an improvement in decision satisfaction. This could be attributed to a couple of factors. One could be that, in this randomized controlled trial, we did not incorporate a service referral or prompt users to take additional steps to address their depressive symptoms. It is possible that neither of the existing service options aligned with the participants’ service expectations.

Second, participants who used the decision aid were more likely to have a preference for and higher likelihood of using a guided internet-based psychological intervention than face-to-face psychotherapy or be uncertain about their service preference, compared with those who searched for mental health information on their own. These findings are important because they suggest that a decision aid may be effective at increasing the acceptability of a guided internet-based psychological intervention, which is a promising and increasingly popular mode of delivering cost-effective mental health services [36,37,59,60]. As a guided internet-based psychological intervention would likely be incorporated in the health care system as a “new normal” and increased investments in digital health today will yield unprecedented access to guided internet-based psychological interventions [60], it was vital to create an evidence-based decision aid to help people who are undecisive when choosing between a guided internet-based psychological intervention and face-to-face psychotherapy. The use of a decision aid in mental health care systems can provide users with the information and support they need to make informed choices that align more with their preferences and values and, in turn, can lead to improved dissemination of e-services and better access to mental health services.

Third, the decision aid used here also demonstrated its implementation value in addition to its impact. The aid was fully automated, which means users can receive its benefits with no additional labor cost. In addition, the aid could be easily incorporated into any e-mental health platform that provides guided self-help or psychotherapy. Furthermore, it is noteworthy that 58 of 71 (82%) participants were able to complete their use of the decision aid within 10 minutes. This is significant because the acceptability of a decision aid among individuals in distress is influenced by factors such as having a reasonable amount of information and ease of use [55]. Last, the positive effect on decision conflict was also independent of depression severity, which indicates its potential for general application across a range of depressive conditions.

### Limitations and Future directions

There are several limitations of this study that are worthy of attention. First, the study sample was mostly women, which may limit the generalizability of the findings. Although most psychological studies have faced a similar issue of recruiting mostly women, future studies should attempt to recruit a more diverse sample to enhance generalizability. Second, this study focused on guided internet-based psychological interventions and face-to-face psychotherapy for depression management without the inclusion of medication. Although this focus aligns with the NICE guideline’s recommendation regarding not routinely offering antidepressant medication as first-line treatment for less severe depression unless that is the person's preference, the current version of the decision aid precludes medication as an option to users. Future studies may consider incorporating information on medication and other treatment options (eg, combined treatments) to provide a more comprehensive, albeit more complex, decision-making tool for individuals with depression. One notable issue of this study is the borderline significant difference in the history of psychological treatment between groups. This indicates a potential trend, and we cannot confidently assert that there is no meaningful difference in prior treatment experiences. Future studies with larger sample sizes are needed to clarify this relationship and adequately assess the impact of prior psychological treatment on study outcomes. Finally, the study did not incorporate behavioral outcomes, such as actual uptake and adoption of a psychological intervention, for the decision aid. Although it is sensible that users may not immediately book a psychological intervention upon completion of the decision aid, future studies may consider including such measures to better understand the impact of decision aids on service uptake. Moreover, health decisions, especially for potentially chronic conditions such as depression, could be an ongoing process. Therefore, it is worth conducting further studies to explore how the decision aid can be integrated into long-term decision-making processes, determine how the decision aid can fit real-life decision-making, and assess the sustainability of the decision aid's effects over an extended period.

### Conclusion

The findings of this study suggest that a multifaceted digital decision aid integrating service users’ psychoeducation about depression and related treatments and preference elicitation can potentially be a useful tool to enhance decision-making. Our online decision aid was not designed to promote guided internet-based psychological interventions but to facilitate making informed choices. Nonetheless, the findings showed that the use of decision aid improved the likelihood of adopting digital treatments, indicating the potential benefits of the decision aid for disseminating digital mental health services in a more user-centered manner that does not involve direct promotion. Service providers may consider incorporating a digital decision aid to support service users to make informed choices and reduce their decision conflict between formats of services.

## Appendix

Multimedia Appendix 1 CONSORT-eHEALTH checklist (V1.6.1).

## Abbreviations

DCS: Decisional Conflict Scale
NICE: National Institute for Health and Care Excellence
OR: odds ratio
PHQ-9: Patient Health Questionnaire-9

## Data Availability

The data that support the findings will be available upon reasonable request from the corresponding author.
